# Mechanical Insights into the Distinct Effects of Ovariectomy Versus Adrenalectomy on Age-Related Thymic Atrophy in Female Mice

**DOI:** 10.3390/ijms27021022

**Published:** 2026-01-20

**Authors:** Junan Chen, Xudong Zhou, Ling Wei, Zixuan Tian, Haozhe Zeng, Fei Yan, Junhua Zhou, Xianyin Zeng, Fengyan Meng, Xiaohan Cao, Haozhou Li, Xingfa Han

**Affiliations:** College of Life Science, Sichuan Agricultural University, Ya’an 625014, China; 15320030117@163.com (J.C.); li1908496536@163.com (X.Z.); 15082471906@163.com (L.W.); 17820733743@163.com (Z.T.); 17858424481@163.com (H.Z.); 17380878829@163.com (F.Y.); lihaidebabayyds@gmail.com (J.Z.); xyzen1966@163.com (X.Z.); mfy0407@126.com (F.M.); caoxiaohan@sicau.edu.cn (X.C.); haozhouli@sicau.edu.cn (H.L.)

**Keywords:** thymic atrophy, ovariectomy, adrenalectomy, fat metabolism, transcriptomics, mice

## Abstract

Age-related thymic atrophy, a hallmark of immunosenescence linked to age-related diseases, involves gonadal and adrenal steroid hormones, but their distinct roles and mechanisms in this process remain unclear. Through biochemical, histological, and RNA-seq analyses, we comprehensively explored the mechanisms underpinning age-related thymic atrophy in response to ovariectomy (OVX) versus adrenalectomy (ADX) in female mice. Compared to the sham controls, OVX overtly ameliorated age-related thymic atrophy, as evidenced by increased thymus mass, a larger gross thymus area, and denser cortex cellularity. In contrast, ADX evidently accelerated age-related thymic atrophy, characterized by increased adipose infiltration and decreased cortex/medulla ratio, obscure cortico-medullary junctions, and sparser thymic cortical cells. Unexpectedly, combined OVX and ADX displayed a more pronounced effect than OVX alone in ameliorating age-related thymic atrophy. Mechanistically, OVX decreased while ADX increased the circulating 17β-estradiol levels in female mice, which drove these opposing outcomes potentially by promoting *Pparg*-mediated thymic fat deposition and blocking *Cdk1*-dependent thymocyte cell cycle progression. Although OVX eliminated gonadal 17β-estradiol production, it appeared to trigger a compensatory adrenal-dependent estrogen biosynthesis, whereas combined OVX and ADX nearly eliminated all estrogen sources, thus leading to a more pronounced effect than OVX alone in ameliorating age-related thymic atrophy in female mice. Notably, OVX increased while ADX decreased serum corticosterone levels, but these alterations exerted minimal impacts on age-related thymic atrophy, highlighting a pivotal role of estrogens over glucocorticoids in accelerating age-related thymic atrophy in females. Undesirably, although OVX ameliorated age-related thymic atrophy, it appeared to simultaneously increase autoimmune susceptibility by downregulating thymic *Cd74* expression. Taken together, our results indicate that OVX ameliorates while ADX accelerates age-related thymic atrophy in females. Estrogens rather than glucocorticoids act as the predominant regulator of this process, potentially via promoting *Pparg*-dependent fat deposition and blocking *Cdk1*-dependent thymocyte cycle progression. However, OVX-induced estrogen depletion also elevated autoimmune risk, emphasizing the need to balance benefits and risks in regulating thymic aging.

## 1. Introduction

The thymus, serving as a pivotal organ in the immune system, plays a central role in the development and maturation of T lymphocytes [1]. Undesirably, the thymus is the most rapidly aging tissue in the body, with progressive atrophy beginning as early as birth [2]. This atrophy is characterized by a reduction in the number of thymocytes and overall thymic cellularity, as well as the infiltration of adipose tissues [2]. Age-related thymic atrophy has significant impacts on immune function, leading to immunodeficiency [3], increased risk of various diseases [4,5], and reduced vaccination efficacy [6]. Thus, understanding the mechanisms underlying age-related thymic atrophy is essential for the development of strategies to promote healthy aging and improve immune function in the elderly. However, the factors and their underpinning mechanisms driving age-related thymic atrophy remain largely elusive.

Age-related thymic atrophy is a complex process involving the intricate interplay of hormonal, cellular, and molecular factors that culminates in reduced output of naïve T cells and subsequent skewing of the immune repertoire [7]. Among the diverse range of endogenous hormones, both gonad-derived sex steroid hormones [8] and adrenal gland-derived glucocorticoid hormones [9] are reported to be strikingly implicated in the initiation and/or progression of thymic atrophy during aging. Several studies have revealed that ablation of sex steroids can induce profound thymic rejuvenation, characterized by increased thymic size, cell number, and immune function [10], whereas in vivo administration of sex steroids significantly inhibits thymus growth and reduces thymic lymphocyte subsets in both male and female rats [10]. Similarly, glucocorticoids have also been reported to exert a major effect on thymic atrophy by inhibiting double-positive (DP) thymocyte proliferation [11] and promoting thymocyte apoptosis [11,12,13]. Additionally, removing the source of glucocorticoids by adrenalectomy can ameliorate thymic atrophy by inhibiting thymocyte apoptosis [13]. Therefore, intervening in the secretion of steroid hormones from both the gonads and adrenal glands appears to be a viable strategy for ameliorating age-related thymic atrophy.

However, it remains unclear whether interventions targeting gonadal and adrenal function exert similar or distinct mechanisms in regulating age-related thymic atrophy. Furthermore, the gonads and adrenal glands share a set of steroidogenic enzymes [14]. The adrenal glands not only produce glucocorticoid hormones but also sex steroid hormones [14]. Most importantly, the gonads and adrenal glands have a complex and bidirectional relationship in terms of hormone synthesis and secretion [15]. Interventions targeting gonadal steroid secretion may, thus, affect the secretion of adrenal glucocorticoids and sex hormones [15], and vice versa. However, most previous studies have only focused on the isolated effects of either the deprivation of gonadal steroid hormones or deprivation of adrenal glucocorticoids on age-related thymic atrophy, without considering their comprehensive and potential interactive impacts. Therefore, this study focused on investigating the effects of ovariectomy (OVX) versus adrenalectomy (ADX), as well as their combination, on age-related thymic atrophy. Through comprehensive research, we found that chronic OVX can ameliorate age-related thymic atrophy, whereas chronic ADX unexpectedly accelerates age-related thymic atrophy. Mechanistically, the opposite secretion profile of estrogens, but not glucocorticoids, between OVX and ADX mice exerts a predominant role in mediating their distinct responses to age-related thymic atrophy in female mice.

## 2. Results

### 2.1. Chronic OVX Delays but Chronic ADX Accelerates Age-Related Thymic Atrophy in Female Mice

**Figure 1:**
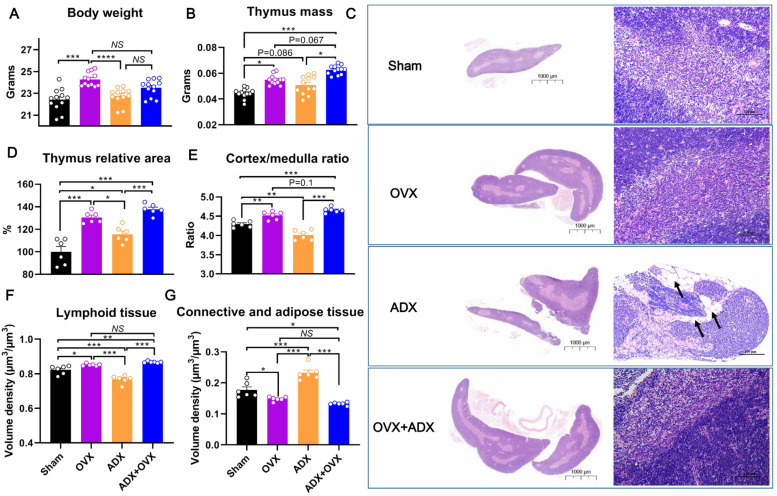
Effects of ovariectomy (OVX), adrenalectomy (ADX), and their combined treatment (ADX + OVX) on body and thymus mass in female mice: (**A**) Body mass (grams) across different treatment groups. (**B**) Thymus mass (grams) across different treatment groups. (**C**) Representative H&E-stained histological images of thymus sections from different treatment groups. Arrows indicate adipocyte infiltration into the thymus; Scare bar (for left images) = 1000 µm; Scare bar (for right images) = 100 µm. (**D**) The thymus area relative to that of sham-operated control mice. (**E**) Ratio of cortex/medulla was quantified using Image J software (Office 2021). (**F**) The volume density of thymic lymphoid tissue. (**G**) The volume density of thymic connective and adipose tissue. Data are presented as mean ± SEM. * *p* < 0.05, ** *p* < 0.01, *** *p* < 0.001, **** *p* < 0.0001, and NS for not significant.

To explore the effects of OVX versus ADX on thymus remodeling and atrophy during aging, female mice were subjected to ovariectomy (OVX), adrenalectomy (ADX), or combined ovariectomy and adrenalectomy (OVX + ADX) at 6 weeks of age, and then their body weight and thymus mass were assessed at 30 weeks of age. Compared to the sham controls, female mice exhibited a marked increase in body weight (*p* < 0.05) following OVX, whereas body weight remained almost unchanged (*p* > 0.05) following either ADX or OVX + ADX (Figure 1A). At decapitation, compared to the sham controls, the thymus mass in female mice increased (*p* < 0.05) following OVX. Additionally, a trend toward a further increase in thymus mass was observed in the OVX + ADX group, though this did not reach statistical significance (*p* = 0.067; Figure 1B). Similarly, the thymus mass in female mice increased (*p* = 0.086) following ADX, and further increased (*p* < 0.05) following ADX + OVX as well (Figure 1B). These results indicate that, if using thymus mass as a hallmark for evaluating thymic atrophy, both OVX and ADX seem to delay age-related thymic atrophy. Additionally, OVX and ADX obviously exert a distinct or additive effect on relieving age-related thymic atrophy in female mice, as evidenced by a heavier thymus mass in OVX + ADX mice than in either the OVX or ADX mice (Figure 1B).

Histological analysis using hematoxylin–eosin (HE) staining was further performed to evaluate thymus morphological changes. In parallel with the increased thymus mass, both OVX and OVX + ADX mice increased (*p* < 0.001) gross thymus area relative to both the sham and ADX mice (Figure 1C,D). Additionally, ADX mice also showed a relatively larger (*p* < 0.05) thymus area than the sham mice (Figure 1C,D). Morphologically, the thymus from the sham control group exhibited obscure cortico-medullary junctions and reduced cellularity in the cortical regions. Compared to the sham group, the thymus of the OVX group displayed denser cellularity in both the cortical and medullary regions, as well as clearer cortico-medullary junctions, indicating that OVX can efficiently delay age-related thymic atrophy (Figure 1C). In contrast, the thymus of the ADX group exhibited adipocyte infiltration (as marked by arrows), an indistinct cortex-to-medulla boundary, and sparser thymic cortical cells (Figure 1C). These histological changes are all typical hallmarks of thymic atrophy, suggesting that ADX accelerates—rather than delays or ameliorates—age-related thymic atrophy. Unexpectedly, the thymus of the OVX + ADX group exhibited more “youthful” structural characteristics compared to the other three groups (Figure 1C), with denser cortical cellularity and more distinct cortico-medullary junctions. Further quantitative analysis revealed that both OVX and OVX + ADX increased (*p* < 0.01) the thymic cortex/medulla ratio, whereas ADX decreased (*p* < 0.05) this ratio compared to sham control mice (Figure 1E). Moreover, the thymic cortex/medulla ratio in OVX + ADX mice tended to be higher than in OVX mice, although this was not statistically significant (*p* = 0.1). This phenomenon suggests that the “regenerative” effect of OVX on the thymus completely counteracts the negative impact of ADX and may even produce a synergistic effect, leading to a more rejuvenated thymic appearance. Stereological analysis confirmed that, compared to the sham controls, ADX markedly decreased (*p* < 0.05) the volume density of functional thymic tissue (i.e., lymphoid tissue) but increased (*p* < 0.001) the volume density of inter-lobular connective and adipose tissue in female mice (Figure 1F,G). In contrast, both OVX and OVX + ADX substantially increased (*p* < 0.05) the volume density of lymphoid tissue, and decreased (*p* < 0.05) the volume density of connective and adipose tissue (Figure 1F,G). Although statistical significance was not attained, the volume density of lymphoid tissue was moderately higher in OVX + ADX mice than in OVX mice (Figure 1F,G).

To confirm whether the short transient slowdown period of liver growth occurs during adrenarche in young female rats, we quantified the mRNA expression of adrenal cytochrome P450 17A1 (*Cyp17a1*), the determinant factor for adrenarche onset [11], and serum dehydroepiandrosterone (DHEA) concentrations during 2–3 wk of age in female rats. Resultantly, the mRNA expression of *Cyp17a1* in adrenal glands markedly increased and peaked (*p* < 0.001) at 3 wk of age, then quickly dropped (*p* < 0.001) to almost undetectable levels at 4 wk of age (Figure 1D). In parallel, serum concentrations of DHEA markedly increased and peaked (*p* < 0.001) at 3 wk of age, and then dropped (*p* < 0.01) to low levels at 4 wk of age (Figure 1E). Therefore, liver growth undergoes a transient slowdown period of growth during adrenarche in young female rats.

Collectively, these results demonstrate that chronic OVX delays age-related thymic atrophy, whereas chronic ADX accelerates rather than ameliorates this atrophy process in female mice. Notably, combined OVX and ADX exerts a synergistic effect, leading to a more pronounced rejuvenation of thymic architecture.

### 2.2. OVX and ADX Cause Opposite Steroid Hormone Secretion Response in Female Mice

**Figure 2:**
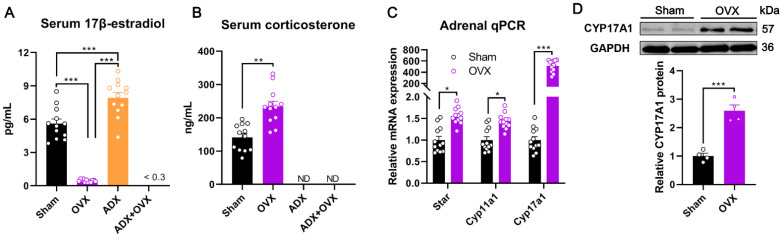
Effects of ovariectomy (OVX), adrenalectomy (ADX), and their combined treatment (ADX + OVX) on serum 17β-estradiol and corticosterone levels in female mice: (**A**) Serum 17β-estradiol levels (pg/mL). (**B**) Serum corticosterone levels (ng/mL). (**C**) The mRNA expression levels of adrenal estrogen biosynthetic enzyme-encoding genes. (**D**) The adrenal CYP17A1 protein expression levels in female mice. Data are presented as mean ± SEM. * *p* < 0.05, ** *p* < 0.01, *** *p* < 0.001.

The steroid hormones secreted by both the ovaries and adrenal glands play important roles in modulating thymus remodeling and atrophy. In particular, ovary-derived 17β-estradiol [16] and adrenal gland-derived corticosterone [17] are both reported to be the primary mediators in accelerating age-related thymic atrophy. To clarify how OVX and ADX affect these circulating steroid hormone levels in female mice, we then assayed the serum concentrations of 17β-estradiol and corticosterone. Resultantly, compared to the sham controls, serum concentrations of 17β-estradiol in female mice were largely eliminated (*p* < 0.001) following OVX, but markedly increased (*p* < 0.001) following ADX by 41.42% (Figure 2A). Interestingly, although serum 17β-estradiol concentrations largely declined in female mice following OVX, it was still detectable in OVX mice but not in OVX + ADX mice using highly sensitive gas chromatography–tandem mass spectrometry (Figure 2A), implicating a compensatory role of the adrenal glands in facilitating 17β-estradiol production when ovarian function is lost. Compared to the sham controls, serum concentrations of corticosterone decreased (*p* < 0.01) to undetectable levels in female mice following ADX, but increased (*p* < 0.01) following OVX (Figure 2B). Meanwhile, serum concentrations of corticosterone in ADX and OVX + ADX mice were undetectable (Figure 2B), indicating no compensatory role for the ovaries to produce corticosterone after adrenal dysfunction.

To further verify the adrenal compensatory role for estrogen biosynthesis following gonadal dysfunction, we used qPCR to assess the expression changes in adrenal key steroidogenic enzyme-encoding genes in female mice following OVX. As anticipated, compared to sham controls, mRNA expression levels of key estrogen-synthesizing genes, including *Star*, *Cyp11a1*, and *Cyp17a1*, were all markedly upregulated (*p* < 0.05) in the adrenal glands of OVX mice (Figure 2C). Using Western blotting, we validated that the CYP17A1 protein—which otherwise is not expressed in the adrenal gland of rodent animals—was expressed in the adrenal glands of mice following OVX (Figure 2D). Additionally, we also detected the protein expression of CYP19A1, another key enzyme critical for estrogen biosynthesis in the adrenal glands of female mice, but no expression was detected either in sham or OVX mice. The restored expression of adrenal CYP17A1 could facilitate the biosynthesis of steroid precursors (e.g., dehydroepiandrosterone), but the absence of CYP19A1 completely restricts adrenal estrogen production. These results indicate that the adrenal glands supply steroid precursors for estrogen synthesis in OVX mice in a compensatory manner, yet cannot complete the full process due to the lack of CYP19A1.

### 2.3. Transcriptomic Responses of Thymus to OVX Versus ADX in Female Mice

**Figure 3:**
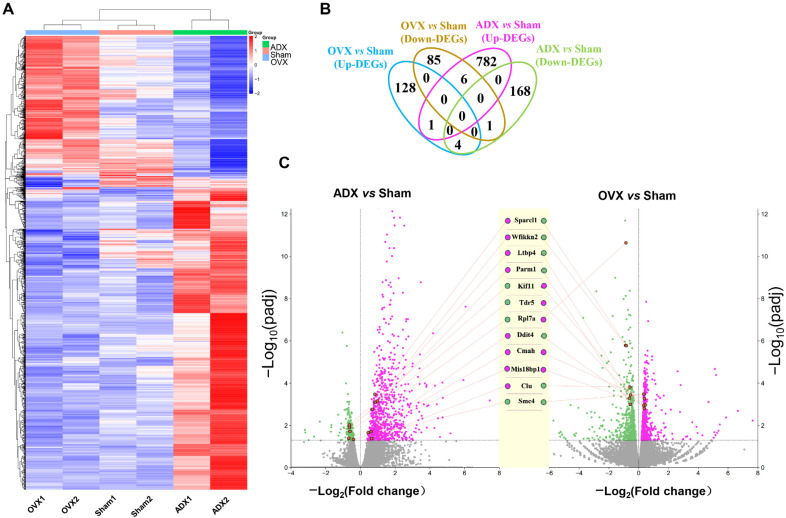
Distinct thymic gene expression patterns in female mice in response to OVX versus ADX: (**A**) Hierarchical clustering heatmap of RNA-seq data showing gene expression patterns in thymus samples from sham, OVX, and ADX female mice. (**B**) Venn diagram illustrating the overlap of DEGs among the OVX vs. sham, ADX vs. sham comparisons. (**C**) Volcano plot analysis highlighting the 12 DEGs shared in OVX vs. sham and ADX vs. sham.

To gain insight into the distinct responses of age-related thymic atrophy to OVX versus ADX, we performed RNA sequencing (RNA-seq) on thymic tissues from the sham control, OVX, and ADX female mice. Hierarchical clustering comparing the patterns of gene expression revealed a clear separation between the OVX and sham, ADX and sham, and notably between the OVX and ADX groups (Figure 3A). Very intriguingly, the gene expression patterns in OVX versus ADX mice were nearly opposite (Figure 3A). These results indicate that OVX and ADX exert distinct or even opposite effects on age-related thymic atrophy in female mice.

Pairwise comparisons among groups identified 3125 differentially expressed genes (DEGs; Figure 3B; see Materials and Methods Section for criteria). Among these, 225 DEGs were detected between the OVX and sham groups, with 127 genes (56%) upregulated and 98 genes (44%) downregulated (Supplemental File S1). In contrast, the number of DEGs detected between the ADX and sham groups was approximately four times more (961 DEGs), with 789 genes (82.1%) upregulated and 172 genes (17.9%) downregulated (Supplemental File S1). Interestingly, the Venn diagram analysis displays only 12 shared DEGs in the OVX vs. sham and ADX vs. sham comparisons (Figure 3C). Moreover, 9 out of these 12 (75%) shared DEGs exhibited opposite expression patterns in response to OVX versus ADX (Figure 3C). These results further support the notion that OVX and ADX induce distinct or opposing age-related thymic atrophy responses in female mice.

### 2.4. Functional Changes in the Thymus in Response to ADX

**Figure 4:**
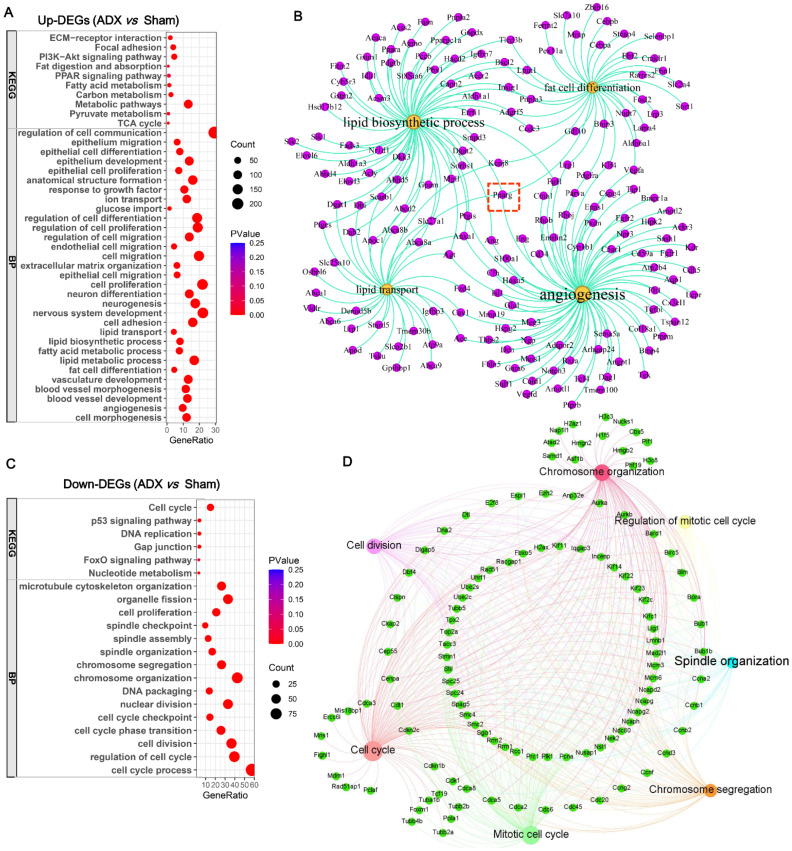
Functional enrichment analysis of differentially expressed genes (up-DEGs) in ADX versus sham groups: (**A**) Functional enrichment of up-DEGs using DAVID (v2024q4). (**B**) Network analysis identified key terms and key up-DEGs involved in thymic adipose infiltration in ADX female mice. Note, Pparg (marked by dotted square) plays a central role in mediating ADX to promote thymic adipose infiltration by enhancing these biological processes. (**C**) Functional enrichment of down-DEGs using DAVID (v2024q4). (**D**) Network analysis identified key terms and key down-DEGs involved in cell cycle blockage in ADX in female mice.

To gain insight into the mechanisms by which ADX accelerated age-related thymic atrophy, functional enrichment analysis of the differentially expressed genes (DEGs) detected between the ADX and sham groups was performed using DAVID (v2024q4) [18]. Resultantly, compared to the sham controls, the upregulated DEGs in the ADX group were predominantly annotated into the biological processes (BPs) of cell proliferation, regulation of cell proliferation, lipid biosynthetic process, fat cell differentiation, lipid transport, blood vessel development, neurogenesis, cell migration, epithelial cell proliferation/development, extracellular matrix organization, and cell morphogenesis, etc., and into the KEGG pathway of metabolic pathways, cell proliferation (e.g., PI3K-Akt signaling pathway), fatty acid metabolism, fat digestion and absorption, the PPAR signaling pathway, extracellular matrix remodeling (e.g., focal adhesion and ECM–receptor interaction), etc. (Figure 4A; Supplemental File S2). These results indicate that ADX triggered the proliferation of certain cell types in the thymus. In particular, ADX induced adipocyte infiltration into the thymus of female mice, primarily by enhancing thymic fat cell differentiation, the lipid biosynthetic process, and lipid transport and angiogenesis (Figure 4B). Notably, *Pparg* plays a pivotal role in regulating these four processes (marked by a dotted square), implicating its central role in mediating ADX to trigger adipose infiltration into the thymus. In contrast, the downregulated DEGs in the ADX group were mainly enriched into the BPs of cell cycle, cell division, cell cycle regulation, chromosome organization, spindle organization, and spindle checkpoint, etc., and into the KEGG pathways of the cell cycle, DNA replication, and the p53 signaling pathway (Figure 4C; Supplemental File S2). The key genes mediating ADX to suppress thymic cell cycle progression were further isolated, as shown in Figure 4D. These results indicate that ADX exerts complex effects on thymic physiology and function. Namely, it promotes proliferation in specific thymic cell populations while inhibiting proliferation in others by blocking their cell cycle progression.

To clarify which cell types were promoted or inhibited by ADX, we first examined the expression profiles of specific cell marker genes identified by previous single-cell RNA-seq (scRNA-seq) studies in thymus or adipose tissues. Notably, compared to the sham control and OVX groups, ADX significantly upregulated adipocyte markers [19] while downregulating markers for double-negative (DN), double-positive (DP), CD4^+^ single-positive (CD4^+^), and CD8^+^ single-positive (CD8^+^) T cells [20] in the thymus of female mice (Figure S1A in Supplemental File S3). This suggests that ADX may increase the proportion of thymic adipocytes while decreasing the number of thymocytes. To validate this, we used the machine learning tool CIBERSORTx [21] to further deconvolute our bulk RNA-seq data. Thymic adipocyte percentages in ADX mice were 2.6-fold and 3.0-fold higher than in sham controls and OVX mice, respectively, while CD4^+^ lymphocyte percentages were slightly reduced (Figure S1B in Supplemental File S3). Additionally, gene set enrichment analysis (GSEA) confirmed that ADX promoted thymic adipogenesis (Figure S1C). Quantification of thymic triglyceride (TG) content further verified that ADX significantly increased thymic TG deposition (Figure S1D). All these results imply that ADX may cause fat deposition into the thymus of female mice. Since increased lipid/adipocyte deposition is a typical hallmark of thymus aging [22], the increased thymic mass in ADX mice does not seem to reflect delayed atrophy but rather increased fat deposition.

**Figure 5:**
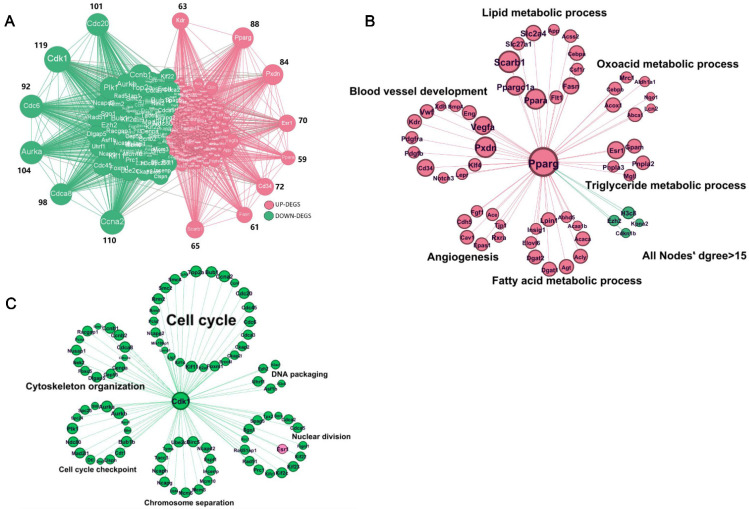
Integrated analysis reveals *Pparg* and *Cdk1* as key mediators of thymic dysfunction in ADX female mice: (**A**) Protein–protein interaction (PPI) network analysis of all upregulated (red) and downregulated (green) genes by ADX in female mice. *Pparg* and *Cdk1* exhibited the highest degree of connectivity (number of first-degree connections) among the upregulated and downregulated genes, respectively. (**B**) Subnetwork of DEGs centered on *Pparg* (nodes with an out-degree > 15). (**C**) Subnetwork DEGs centered on *Cdk1* (green node) (nodes with an out-degree > 15). Functional enrichment analysis of DEGs was conducted by DAVID (v2024q4).

To elucidate the molecular mechanism by which ADX promoted lipid/adipocyte deposition and, thus, accelerated thymic aging in females, we performed protein–protein interaction (PPI) network analysis using all ADX-induced DEGs in the thymus to identify the hub genes mediating this process. Among upregulated hub genes, Pparg showed the highest number of connected nodes (Figure 5A). As a key transcription factor essential for adipogenesis and lipogenesis [23], Pparg-centered nodes (out-degree > 15) were isolated and used to construct a subnetwork (Figure 5B) for further analysis. As expected, most Pparg-centered key genes were upregulated by ADX (Figure 5B), with functional enrichment into adipogenesis and lipid metabolism (e.g., *Scarb1*, *Csf1r*, *Cebpa*, *Slc27a1*, *Acss2*, *Flt1*, *Insig1*, and *Pdgfb*), as well as angiogenesis (Vegfa, Vwf, Cdh5, and Kdr) (Figure 5B; Supplemental File S4). Notably, angiogenesis is critical for adipose tissue development, as lipid synthesis depends on vascular support [24]. These results suggest that ADX may exacerbate thymic lipid and adipocyte deposition primarily by activating Pparg signaling, thereby stimulating both adipogenesis and angiogenesis. Among the downregulated genes, *Cdk1* emerges as the central hub gene with the highest number of connected DEG nodes (Figure 5A). As a well-known key regulator of cell cycle progression [25], *Cdk1*-centered DEGs (out-degree > 15) were further isolated for subnetwork construction (Figure 5C). Most of these *Cdk1*-centered DEGs were significantly downregulated in ADX mice, with functions enriched in mitotic cell cycle processes, cell proliferation, cell division, and metabolic processes (Figure 5C; Supplemental File S4). Collectively, ADX accelerates age-related thymic atrophy in females through dual mechanisms. Namely, it activates Pparg signaling to promote lipogenesis/adipogenesis and fat deposition, while downregulating Cdk1 to block thymocyte cell cycle progression, thereby accelerating thymus degeneration and atrophy during aging.

Glucocorticoid and estrogen hormones exert physiological actions by binding to their specific receptors, i.e., glucocorticoid receptor (GR) and estrogen receptors (including ERα and ERβ), which are all nuclear transcription factors. To further clarify the causal link between steroid hormone changes following ADX and thymic atrophy in female mice, we screened the aforementioned hub genes for potential glucocorticoid response elements (GRE) and estrogen response elements (ERE) in their promoter regions, spanning 1500 bp upstream and 500 bp downstream of the transcription start site using Ciiider [26]. Notably, nearly all 15 upregulated (*Pparg*, *Vegfa*, *Fasn*, *Ppara*, *Cdh5*, *Sarb1*, *Vwf*, *Col6a1*, *Cd34*, and *Pxdn*) and 15 downregulated (*Cdk1*, *Ccnb1*, *Cdc6*, *Cdc20*, *Top2a*, *Aurka*, *Bub1*, *Mad2l1*, *Kif11*, *Bub1b*, and *Kif2c*) hub genes in the ADX group contain potential ERβ binding sites (Supplemental File S5). In contrast, only a few upregulated (*Pparg*, *Ppara*, *Col6a1*, and *Cd34*) and downregulated (*Cdk1*, *Ccnb1*, and *Kif11*) hub genes harbored GRE (Supplemental File S4). Surprisingly, only a few upregulated (*Ppara*) and downregulated (*Mad2l1* and *Kif2c*) hub genes contain potential ERα binding sites (Supplemental File S5). These findings suggest that elevated estrogens rather than glucocorticoids play a central role in accelerating age-related thymic atrophy in ADX female mice, and that estrogens exert such an effect primarily by binding to and activating ERβ, rather than ERα, signaling. Notably, the most critical upregulated hub gene (*Pparg*) and the most critical downregulated hub gene (*Cdk1*) both contain potential binding sites for ERβ and GR (Supplemental File S5), indicating that estrogens and glucocorticoids may act synergistically or additively to regulate thymic atrophy by co-modulating the expression of these two hub genes.

Taken together, the increase in thymus mass caused by ADX does not reflect a genuine delay in thymic atrophy. This phenomenon is likely attributable to excessive adipose infiltration and deposition, which is caused by the elevation of estrogens and decline in glucocorticoids.

### 2.5. Functional Changes in the Thymus in Response to OVX

**Figure 6:**
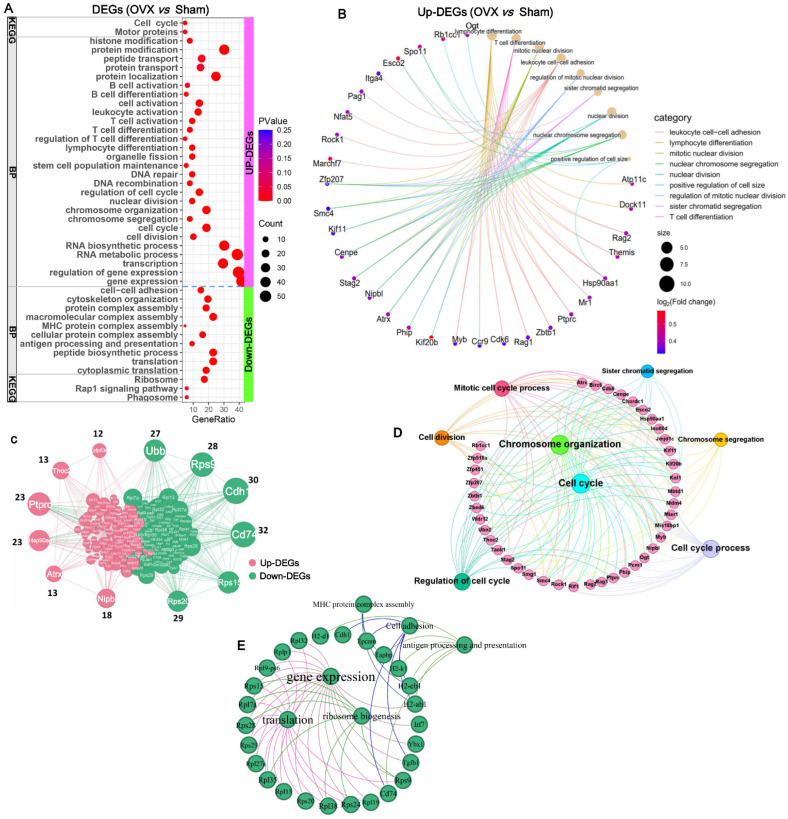
Functional enrichment analysis of differentially expressed genes (DEGs) in OVX versus sham controls: (**A**) Functional enrichment of DEGs using DAVID (v2024q4). (**B**) Network analysis revealed that OVX ameliorated age-related thymic atrophy maintenance through promoting thymocyte proliferation and differentiation. (**C**) Protein–protein interaction (PPI) network analysis of all upregulated (red) and downregulated (green) genes by OVX in female mice. (**D**) Functional enrichment analysis of the upregulated hub DEGs by OVX in female mice. (**E**) Functional enrichment analysis of the downregulated hub DEGs by OVX in female mice. Functional enrichment analysis of DEGs was conducted by DAVID (v2024q4).

To explore the mechanisms by which OVX delayed age-related thymic atrophy, functional enrichment analysis of DEGs in the thymus in the OVX versus sham-operated mice was performed using DAVID. The upregulated DEGs in the OVX group were primarily annotated into the BPs of cell cycle, cell division, cell cycle regulation, chromosome organization, DNA repair, T-cell differentiation, and B-cell differentiation, etc., and into the KEGG pathways of cell cycle and motor proteins (Figure 6A; Supplemental File S2). Consistent with this, OVX increased the expression of cell markers for double-negative (DN), double-positive (DP), CD4^+^ single-positive (CD4^+^), and CD8^+^ single-positive (CD8^+^) T cells compared to sham controls (Figure S1A in Supplemental File S3). These results indicate that OVX delays age-related thymic atrophy by ameliorating the age-associated decline in thymocyte differentiation. Key DEGs involved in these processes are highlighted in Figure 6B. In contrast, the downregulated DEGs in the OVX group were primarily annotated into the BPs of gene expression, translation, antigen processing and presentation, MHC protein complex assembly, and cytoskeleton organization, and into the KEGG pathways of ribosome function and antigen processing and presentation (Figure 6A; Supplemental File S2). Notably, OVX downregulated MHC complex assembly and antigen processing/presentation, which are critical for thymocyte-positive and -negative selection. Concomitantly, OVX reduced the expression of regulatory T cell (Treg) marker genes in the thymus compared to sham controls (Figure S1A in Supplemental File S3). These findings suggest that, while OVX mitigates age-related thymic atrophy, it may enhance autoimmunity by impairing thymocyte negative selection and reducing Treg production, highlighting a trade-off between thymic maintenance and immune tolerance.

To further clarify the mechanisms underlying the increased thymus mass and index following OVX, we performed protein–protein interaction (PPI) network analysis of all DEGs (Supplemental File S1) using STRING. Genes were ranked by the number of first-degree connections, with the top 15 most highly connected genes selected as hub genes for further analysis (Figure 6C). Functional enrichment analysis showed these upregulated hub genes were predominantly enriched in chromosome organization (*Cenpe*, *Nipbl*, *Hsp90aa1*, *Atrx*, *Esco2*, *Kif11*, and *Smc4*), T-cell differentiation (*Ccr9*, *Cdk6*, *Mr1*, *Myb*, *Ptprc*, *Rag1/2*, *Zbtb1*, and *Themis*), and cell cycle-related processes (*Cenpe*, *Nipbl*, *Ptprc*, *Cdk6*, *Atrx*, *Esco2*, *Kif11*, and *Smc4*) (Figure 6D). In contrast, downregulated hub genes were primarily annotated into gene expression, translation, ribosome biogenesis, antigen processing and presentation, and MHC protein complex assembly (Figure 6E).

Using ciiider, we further screened these hub genes for potential ERα/β and GR binding sites in their promoter regions. Notably, nearly all 15 upregulated (*Smc4*, *Smg1*, *Kif11*, *Wdr12*, *Hsp90aa1*, *Thoc2*, *Ptprc*, *Esco2*, and *Atrx*) and 15 downregulated (*Cd74*, *Cdh1*, *Ubb*, *Rps9*, *Rps15*, *Rps20*, *Rps28*, *Rps29*, *Rpl7a*, *Rpl19*, *Rpl27a*, and *Rpl38*) hub genes contain the potential ERβ binding sites, while only the upregulated hub gene *Smc4* harbors a potential ERα binding sequence (Supplemental File S4). These results reinforce our above concept that estrogen-mediated thymic atrophy primarily occurs via ERβ activation. Additionally, only a subset of upregulated (*Kif11*, *Ptprc*, *Esco2*, and *Atrx*) and downregulated (*Cd74*, *Rpl7a*, and *Rpl35*) hub genes contain potential GR binding sites (Supplemental File S4), further supporting the dominance of estrogens over glucocorticoids in regulating thymic atrophy. Of note, the downregulated hub gene *Cd74* contains both potential ERβ and GR binding sites (Supplemental File S4). CD74, also called cluster of differentiation of 74, is central for the assembly and stability maintenance of MHC class II molecules [27]. Deletion of CD74 leads to reduced peptide diversity of MHC class II molecules on the surface of medullary thymic epithelial cells (mTECs), allowing partial escape of high-affinity autoreactive T cells and, therefore, inducing autoimmune diseases [27]. Thus, downregulation of *Cd74* may mediate enhanced autoimmune susceptibility in OVX mice.

Collectively, these results suggest that OVX ameliorated age-related thymic atrophy primarily by promoting and maintaining thymocyte cell cycle processes. In addition, it may also enhance autoimmune susceptibility by impairing negative selection of thymocytes and decreasing production of Treg cells from the thymus via downregulating thymic *Cd74* expression.

### 2.6. Identification of Thymic Atrophy-Connected Gene Modules by Weighted Gene Co-Expression Network Analysis

**Figure 7:**
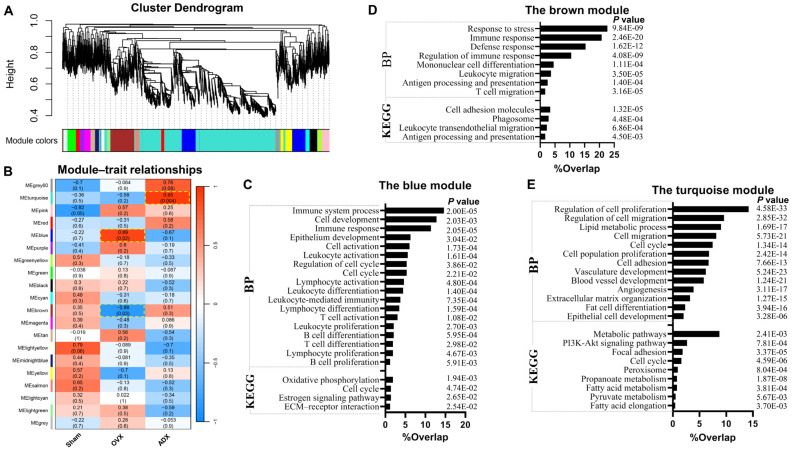
Identification of thymic atrophy-connected gene modules by weighted gene co-expression network analysis (WGCNA): (**A**) Dendrogram of all genes, clustered on the basis of a topological overlap matrix (TOM). Each branch in the clustering tree represents a gene, and co-expression modules were constructed in different colors. (**B**) Module–trait heatmap of the correlation between the clustering gene module and thymic atrophy status. Each module contains the corresponding correlation coefficient and *p* value. (**C**) Functional enrichment analysis of the blue module genes. (**D**) Functional enrichment analysis of the brown module genes. (**E**) Functional enrichment analysis of the turquoise module genes. Functional enrichment analysis of the module genes was conducted by DAVID (v2024q4).

To better understand the molecular mechanisms underlying the age-related thymus atrophy seen in response to OVX versus ADX, we performed weighted gene co-expression network analysis (WGCNA) on the transcriptomic data. Co-expression modules were generated through linking gene expression to thymic atrophy status (Figure 7A). Resultantly, the blue (r = 0.89, *p* = 0.02) and brown (r = −0.86, *p* = 0.03) modules were correlated positively and negatively, respectively, to the delayed age-related thymic atrophy in OVX mice (Figure 7B). Functional enrichment analysis indicates that the positively related blue module genes were predominantly annotated into the BPs of immune system process, cell development, cell cycle, lymphocyte differentiation/proliferation, etc., and into the KEGG pathways of oxidative phosphorylation, cell cycle, and estrogen signaling pathway (Figure 7C), corresponding to delayed age-related atrophy of thymus in OVX mice. Meanwhile, the negatively related brown module genes were annotated into the BPs of immune/defense response, T-cell migration, antigen processing and presentation, etc., and into the KEGG pathways of cell adhesion molecules, phagosome, leukocyte migration, antigen processing and presentation, etc. (Figure 7D), also supporting the view that OVX disrupts thymocyte positive/negative selection. The turquoise module was identified to be positively correlated to (r = −0.95, *p* = 0.004) (Figure 7B) the accelerated age-related thymic atrophy in ADX mice. Functional enrichment analysis shows that the turquoise module genes were mainly enriched into the BPs of lipid metabolic process, fat cell differentiation, angiogenesis, cell migration and proliferation, etc., and into the KEGG pathways of metabolic pathways, the PI3K-Akt signaling pathway, cell cycle, fatty acid/pyruvate metabolism, and fatty acid elongation (Figure 7E). These results also highlight adipocyte infiltration and deposition as critical drivers of ADX-accelerated thymic atrophy.

**Figure 8:**
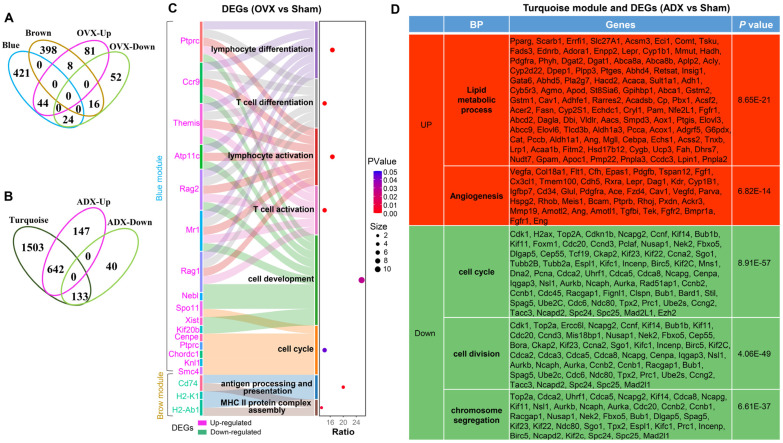
Identification of key module genes mediating thymic atrophy regulation: (**A**) Venn overlap analysis of key module genes connected to thymic atrophy status in OVX mice with OVX-regulated DEGs. (**B**) Venn overlap analysis of key module genes connected to thymic atrophy status in ADX mice and ADX-regulated DEGs. “Ratio” refers to the proportion of key genes enriched in a specific functional term relative to all genes in the module; “Size” denotes the exact number of key genes enriched in a specific functional term. (**C**) Functional enrichment analysis of identified key module genes in OVX mice. (**D**) Functional enrichment analysis of identified key module genes in ADX mice.

Overlap analysis between the blue module genes and OVX-regulated DEGs identified 44 genes significantly upregulated in OVX versus sham controls (Figure 8A). These key genes were predominantly annotated into cell cycle (*Spo11*, *Cenpe*, *Ptprc*, *Chordc1*, *knl1*, and *Smc4*), cell development (*Ptprc*, *Ccr9*, *Themis*, *Atp11c*, *Rag2*, *Mr1*, *Rag1*, *Nebl*, *Spo11*, *Xist*, and *Kif20b*), and T-cell differentiation/activation (*Ptprc*, *Ccr9*, *Themis*, *Rag2*, *Rag1*, and *Mr1*) (Figure 8C). These key genes may play a predominant role in mediating OVX to ameliorate age-related thymic atrophy in female mice. Interestingly, overlap analysis between the brown module genes and OVX-regulated DEGs identified 16 genes significantly downregulated in OVX versus sham controls (Figure 8A). Of those, *Cd74* was involved in both antigen processing and presentation and MHC class II complex assembly (Figure 8C), further highlighting that downregulation of *Cd74* is a critical factor contributing to increased autoimmune susceptibility in OVX female mice. Additionally, the overlap analysis between turquoise module genes and ADX-regulated DEGs identified *Pparg* and *Cdk1*, which serve as key regulators of adipogenesis and the cell cycle, respectively (Figure 8B,D).

**Figure 9:**
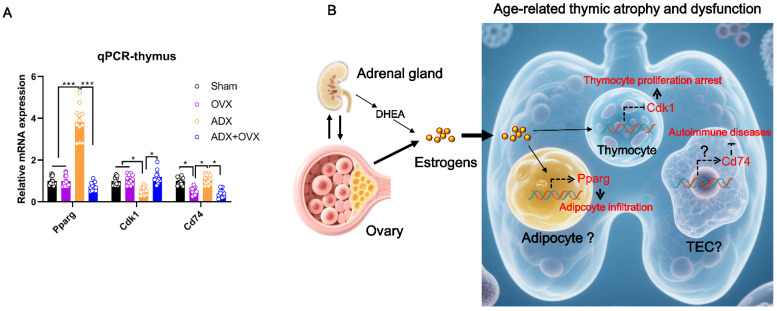
The potential mechanism underpinning the intricate crosstalk between ovaries and adrenal glands in regulating age-related thymic aging in females: (**A**) qPCR validation of the key genes mediating estrogen hormones in regulating age-related thymic atrophy and dysfunction. (**B**) The putative crosstalk between ovaries and adrenal glands in regulating thymic aging. Data are shown as the mean ± SEM, *n* = 12; * *p* < 0.05, *** *p* < 0.001.

We further performed qPCR to confirm the changes in these key genes. Consistent with the RNA-seq data, OVX led to a significant reduction in *Cd74* expression, whereas ADX resulted in upregulated *Pparg* and downregulated *Cdk1* in the thymus (Figure 9A). Based on these findings, we proposed a mechanism involving the intricate crosstalk between the ovaries and adrenal glands in regulating age-related thymic aging in females, as illustrated in Figure 9B. Namely, intricate crosstalk exists between the ovaries and adrenal glands in each other’s hormone production (i.e., estrogens and glucocorticoids). In particular, estrogen hormones directly secreted from the ovaries or indirectly from adrenal glands activate *Pparg* transcription to promote lipogenesis (in adipocytes or other cells) and/or adipogenesis and induce fat deposition into the thymus, meanwhile inhibiting *Cdk1* transcription and thereby potentially blocking thymocyte proliferation and maintenance. These two processes synergistically contribute to accelerated thymic atrophy during aging. However, while reduced estrogen levels in OVX or OVX + ADX mice were paralleled by decreased thymic *Cd74* expression, elevated estrogen levels in ADX mice were not accompanied by increased thymic *Cd74* expression. Thus, it seems that factors other than estrogen mediate OVX/ADX to regulate thymic *Cd74* expression in female mice. Given that thymic epithelial cell (TEC) *Cd74* expression critically regulates autoimmune diseases [27], decreased thymic *Cd74* expression may occur in TECs of OVX mice and thereby enhance autoimmune susceptibility (Figure 9B).

## 3. Discussion

A growing body of evidence has shown that both the gonads and adrenal glands are implicated in the initiation and/or progression of age-related thymic atrophy, primarily through the secretion of steroid hormones [8,9]. However, the distinct roles and mechanisms of gonadal and adrenal dysfunction in regulating age-related thymic atrophy remain largely unclear. Herein, our findings clearly demonstrate that chronic OVX ameliorates age-related thymic atrophy, as evidenced by increased thymus mass and thymic tissue area. In contrast, although chronic ADX was associated with increased thymus mass and thymic tissue area, likely due to substantial lipid and/or adipocyte deposition, it still accelerated age-related thymic atrophy overall. This is supported by an indistinct cortex-to-medulla boundary and sparser thymic cortical cells. Unexpectedly, the combination of OVX and ADX exerts a more pronounced role than OVX alone in ameliorating the age-related thymic atrophy. Mechanistically, OVX decreases while ADX increases, circulating 17β-estradiol levels in female mice. Transcriptomic profiling and integrated data analysis revealed that 17β-estradiol plays a predominant role in promoting thymic adipogenesis and blocking thymocyte cell cycle progression, thereby driving the opposite responses to age-related thymic atrophy in OVX versus ADX mice. Although OVX leads to an increase and ADX causes a decrease in circulating corticosterone levels, these alterations appear to have minor or minimal impacts on age-related thymic atrophy in female mice. Consistent with our findings, previous studies have also reported that OVX-induced thymic enlargement reflects genuine parenchymal expansion, whereas ADX-induced enlargement does not involve functional thymic tissue [28]. Collectively, our results provide novel insights into the intricate crosstalk between gonads and adrenal glands in modulating thymic aging, highlighting a pivotal role of estrogen hormones over glucocorticoids in accelerating thymic atrophy and dysfunction in females.

Indeed, gonadal steroid hormones, including both estrogens and androgens, are well-established as a pivotal driver of thymic aging. For instance, in both female and male rodents, thymic atrophy accelerates after puberty when there is elevation of serum sex steroid hormones [29,30]; conversely, gonadal steroid deprivation via castration elicits robust thymic regeneration [10,30]. Additionally, the administration of sex steroids could substantially induce thymic atrophy and cause immunodeficiency [8,31,32]. Specifically, both in vivo and in vitro studies have further demonstrated that estrogen hormone supplementation promotes thymic atrophy by eliminating early thymic progenitors and inhibiting proliferation of thymocytes [16,31,33]. Thus, the decline in estrogen levels largely accounts for the mitigation of age-related thymic atrophy in mice following OVX. In addition, OVX seemed to trigger adrenal compensation for estrogen production. This was evidenced by the higher 17β-estradiol levels maintained in OVX mice compared to those in OVX + ADX mice. Supporting this compensatory mechanism, both qPCR and Western blotting analyses confirmed the restored expression of CYP17A1, the key enzyme responsible for estrogen precursor (DHEA) production in the adrenal glands of OVX mice. In support, despite the fact that CYP17A1 expression in rodent adrenal glands remains controversial [34,35], some studies convincingly validate the expression of Cyp17a1 at both mRNA and protein levels in rodent adrenal glands [36,37]. This compensatory mechanism well explains why OVX + ADX exerts a more pronounced effect than OVX alone in ameliorating age-related thymic atrophy, as the combined ablation of both glands nearly eliminates all estrogen sources from the female mice. Further studies are warranted to elucidate the mechanisms by which OVX restores CYP17A1 expression in the adrenal glands of mice.

Previous studies have evidenced that chronic or high-dose glucocorticoid exposure exacerbates thymic atrophy, primarily through the induction of thymocyte apoptosis [38]; conversely, eliminating glucocorticoid sources by ADX can ameliorate thymic atrophy by inhibiting thymocyte apoptosis [13]. In our studies, however, OVX increased circulating corticosterone levels while paradoxically ameliorating age-related thymic atrophy. This contradiction is likely because the stimulatory effect of estrogen deprivation on thymocyte proliferation outweighs the pro-apoptotic action of elevated corticosterone, thereby ultimately leading to thymic maintenance. Indeed, in ADX female mice, corticosterone deprivation also failed to counteract the estrogen elevation-induced suppression of thymic maintenance. All these results imply that estrogen hormones play a pivotal role over glucocorticoids in accelerating thymic atrophy and dysfunction in females during aging. It should be emphasized that the duration of ADX appears to be a key factor in determining its effect on thymus atrophy. Previous studies have shown that ADX could ameliorate thymus atrophy at 2 weeks after surgical operation [13], whereas we observed that long-term ADX (i.e., 26 weeks) could accelerate rather than ameliorate age-related thymus atrophy. This is possibly because the ADX-induced compensatory biosynthesis of estrogen requires a certain period of time. In addition, although glucocorticoids are generally regarded as immunosuppressive, evidence indicates they also play positive roles in thymocyte selection and development. For instance, the absence of glucocorticoid signaling in thymocytes results in an immunocompromised state [39]. Furthermore, some studies have even shown that glucocorticoids could delay age-associated thymic atrophy through directly promoting double-negative and single-positive thymocyte proliferation within the thymus [40]. Thus, glucocorticoids appear to exert multifaceted effects on thymic atrophy during aging, which may depend on factors such as their local concentrations in the thymus, the duration and magnitude of exposure, and the physiological context, including interactions with other hormonal signals. More studies are required to evaluate the dose-dependent and time-course effects of glucocorticoids on thymic thymocyte development dynamics, as well as to dissect the crosstalk between glucocorticoid signaling and other endocrine factors (e.g., estrogens) in the regulation of thymic aging.

The two most prominent hallmarks of age-related thymus atrophy are the infiltration of adipose tissues and the progressive loss of thymocytes, which essentially reduce thymic cellularity and impair naïve T-cell production [29]. Notably, with advancing age, adipocytes gradually become the dominant component of the thymic parenchyma and ultimately account for up to 80% of thymic volume in elderly individuals [41]. Consistent with these typical hallmarks of thymic aging, the elevation of estrogens in ADX mice was accompanied with fat infiltration and a loss of thymocytes in the thymus, whereas deprivation of circulating estrogens by OVX, alone or with ADX, reversed those phenotypical changes. These results suggest that the elevation of estrogens plays a pivotal role in mediating ADX to accelerate age-related thymic dysfunction in female mice, primarily by driving thymic adipose infiltration and thymocyte loss. Mechanistically, transcriptomic profiling and integrated data analysis revealed that the elevation of estrogens activated thymic PPARγ signaling. This activation drives thymic adipogenesis, fatty acid biosynthesis [23], and angiogenesis [42], ultimately leading to thymic fat infiltration. Indeed, in breast cancer cells, estrogen hormones can potently activate Pparg transcription, subsequently promoting adipogenesis and angiogenesis to facilitate adipose expansion [43]. On the other hand, we found that estrogens also suppressed thymocyte proliferation, primarily by downregulating Cdk1, a central cell cycle regulator. In support, transcription factor binding motif analysis identified ERβ binding sites in the promoter regions of both Pparg and Cdk1 genes, indicating a direct transcriptional regulation by estrogens. Collectively, these results suggest that ADX-induced elevation of estrogen hormones accelerated age-related thymic atrophy in females, likely by promoting Pparg-dependent adipogenesis and blocking Cdk1-dependent thymocyte cell cycle progression.

Although OVX ameliorated thymic atrophy during aging, it seemed to simultaneously enhance autoimmune susceptibility in females by impairing MHC complex assembly, antigen presentation, and regulatory T-cell (Treg) development. Mechanistically, we found that OVX significantly reduced thymic Cd74 (the MHC class II invariant chain) expression, a central hub gene associated with autoimmune diseases [40]. CD74 mediates MHC class II antigenic peptide loading and presentation, thus playing an essential role in thymocyte-positive and -negative selection [27,44]. Notably, deletion of Cd74 substantially reduces peptide diversity of MHC class II molecules [27], impairs the elimination of autoreactive T cells in the thymus [44], and causes defects in Treg development by downregulating Foxp3 [45], ultimately promoting autoimmune diseases. Collectively, these results may imply that downregulation of thymic Cd74 could contribute to enhanced autoimmune susceptibility in OVX mice, primarily by disrupting the development and function of Treg cells. In support, previous studies have reported that sex hormones modulate Treg cell frequency and function, thereby influencing susceptibility to inflammatory diseases in humans [46]. Thus, the trade-off between thymic maintenance and autoimmune risk emphasizes the necessity for therapeutic strategies targeting thymic atrophy to balance immune reconstitution against autoimmunity.

There are several limitations in the present study. Firstly, our study identified estrogen hormones as a dominant regulator over glucocorticoids in driving age-related thymic atrophy and dysfunction in females, but the specific cell types (e.g., thymic epithelial cells and adipocyte progenitors) mediating estrogen-induced atrophy remain undefined. Future single-cell RNA-seq studies could resolve cell-type-specific effects of estrogen on thymic niche cells. In addition, direct functional assessment of the thymus in ADX + OVX mice treated with estradiol and/or corticosterone is also required. Secondly, we demonstrate that an intricate interplay exists between gonadal and adrenal steroid hormones in regulating thymus aging, but the underlying molecular mechanisms governing this interplay require further elucidation. Thirdly, we observed that OVX triggered adrenal estrogen production through a compensation mechanism, but the specific signaling cascades or transcriptional networks through which OVX stimulates adrenal estrogen precursor production were not addressed and thus warrant further investigation. Fourthly, due to limited tissue availability, this study did not directly evaluate functional changes in the thymus following OVX versus ADX. Additional quantification of thymic cellularity (e.g., naive T-cell export) using flow cytometry is therefore needed. Fifthly, we observed that although OVX ameliorated thymic atrophy during aging, it seemed to simultaneously enhance autoimmune susceptibility in females by downregulating Cd74. However, the underlying mechanism governing this dual effect remains unelucidated and, thus, warrants further investigation. Finally, this study was conducted exclusively in female mice, and the results may not directly translate to humans or male subjects. Given the species- and sex-specific differences in thymic biology and aging processes, caution is warranted when extrapolating these findings.

In conclusion, our study provides novel insights into the distinct roles of OVX versus ADX and the intricate interplay between gonadal and adrenal steroid hormones in regulating age-related thymic atrophy, highlighting estrogen hormones as the dominant regulator over glucocorticoids in driving thymic atrophy and dysfunction in female subjects during aging. Additionally, although OVX ameliorates age-related thymic atrophy, it potentially enhances autoimmune susceptibility in females as well, emphasizing the necessity of balancing the benefits and drawbacks of estrogen depletion on thymic function and exploring more precise regulatory strategies to optimize thymic function during aging.

## 4. Materials and Methods

### 4.1. Animals and Treatment

Six-week-old female C57BL/6J mice were purchased from HuaXi Laboratory Animal Center of Sichuan University (Chengdu, China). The mice were randomly divided into 4 groups, with 12 mice each. All experimental animals were housed four per cage in an animal facility with a controlled 12:12 h light/dark cycle, a temperature of 22 ± 2 °C, and a humidified environment. The mice had free access to a standard commercial mouse chow (Beijing Keao xieli Feed Co., Ltd., Beijing, China) and regular drinking water. The details of the feed composition are available at https://www.keaoxieli.com/product/137.html (accessed on 26 November 2023). For adrenalectomy (ADX) and/or ovariectomy (OVX), dorsal incisions of the skin and muscle layers were made on both sides of the mice under anesthesia, the blood vessels supplying both adrenals were cauterized to prevent bleeding, and subsequently the entire adrenal glands and/or ovaries were removed [47]. Mice in the sham-operated group underwent the same procedure, except for cauterization and removal of adrenal glands or ovaries. All ADX and OVX + ADX mice were provided with 0.9% saline to maintain body salt balance.

### 4.2. Sample Collections and Parameter Measurements at Decapitation

At 30 weeks of age, all mice were anesthetized with isoflurane (Fluriso; VetOne, Boise, ID, USA) and then euthanized by decapitation. To minimize the influence of estrous cyclicity on the experimental results, all mice from the sham and ADX groups were sacrificed during the diestrus phase. Trunk blood was collected and centrifuged at 2000× *g* for 15 min at 4 °C, and the sera were stored at −20 °C until analysis. After sacrifice, the thymus was collected and weighed, and each thymus was divided into four portions. Three portions were immediately frozen in liquid nitrogen and stored at −80 °C for RNA sequencing, qPCR, and thymus triglyceride content analysis; the remaining one was fixed in 10% buffered formalin and used for histological analysis.

### 4.3. Serum Hormone and Thymus Triglyceride Assay

Serum corticosterone (ADI-900-097, Enzo Life Sciences, Ann Arbor, MI, USA) was determined with commercial ELISA kits, according to the manufacturer’s instructions. Serum 17β-estradiol was assayed using highly sensitive gas chromatography–tandem mass spectrometry, as previously described [48]. The triglyceride (TG) content in the thymus was assayed using a TG assay kit (A110-1-1, Nanjing Jiancheng Bioengineering Institute, Nanjing, China) according to the manufacturer’s recommended protocol. The TG content in thymus tissues was normalized by protein concentrations, which were determined using a BCA protein assay kit (Shenneng Bocai Biotechnology Co., Ltd., Shanghai, China).

### 4.4. Hematoxylin–Eosin Staining

Following the fixation of thymus with a 10% buffered formalin solution for approximately 48 h at room temperature, thymus tissues were dehydrated in ethanol and then embedded in paraffin wax, sectioned (5 μm), and stained with hematoxylin and eosin (H&E) (G2161 Solarbio, Beijing, China) for light microscopy. Morphometric analysis was performed to objectively measure alterations in the cortical and medullary sizes of the thymus. The ratio of cortex to medulla was calculated as previously described [49] using the Image J image processing and analysis software (Bethesda, MD, USA). Stereological analyses were further performed to quantify the volume densities of thymic functional tissue (i.e., lymphoid tissue) and inter-lobular connective and adipose tissue, following the method detailed in Plećas-Solarović et al. (2006) [50].

### 4.5. RNA Sequencing

Total RNA was extracted from thymus tissue samples using TRIzol™ Reagent (Invitrogen, Waltham, MA, USA). Thymi from six mice were pooled together as one biological replicate, and two biological replicates in each group were used. The RNA samples were then prepared with the RNA Nano 6000 Assay Kit for Bioanalyzer 2100 System (Agilent Technologies, Santa Clara, CA, USA) to determine the quantity and quality of RNA. A total of 1000 ng of RNA per sample was used as input material, with RNA integrity numbers (RINs) greater than or equal to 8.5. mRNA was subsequently purified from the total RNA using oligo (dT) magnetic capture beads. The libraries were synthesized using the NEBNext UltraTM RNA Library Prep Kit for Illumina (NEB, Ipswich, MA, USA), following the manufacturer’s instructions. Subsequently, the cDNA library underwent sequencing on the Illumina NovaSeq platform to obtain 150 bp of paired-end sequence. To generate clean reads, Fastp (version 0.19.7) was used to filter out low-quality reads (defined as those with more than 50% bead scores Qphred ≤ 20) and to remove adapter sequences. The gene expression level was then calculated using the reads-per-kilo-bases-per-million-reads (RPKM) method. Pairwise comparisons between groups were performed using the DESeq2 R package (1.16.1). The resulting *p*-values were adjusted using Benjamini and Hochberg’s approach for controlling the false discovery rate. Genes with an adjusted *p* value  <  0.05 were assigned as differentially expressed (DEGs).

### 4.6. Functional Enrichment Analysis

The gene ontology (GO) and the Kyoto Encyclopedia of Genes and Genomes (KEGG) analysis were performed using the bioinformatics Database for Annotation, Visualization, and Integrated Discovery (DAVID) (v2025_1). A significance threshold of *p* value < 0.05 was set for all GO terms and KEGG pathways. Gene Set Enrichment Analysis (GSEA) was performed using GSEA (v4.3.2) software. Genes, including all DEGs and non-DEGs, were pre-ranked based on the −log10 (*p*-value) multiplied by the sign of gene log2 Fold Change, such that the upregulated genes had positive scores and downregulated had negative scores. This application scores a sorted list of genes with respect to their enrichment of selected functional categories (Gene Ontology [GO], Kyoto Encyclopedia of Genes and Genomes [KEGG] and Reactome). Terms annotating more than 500 or fewer than 5 genes were discarded. The significance of the enrichment score was assessed using 1000 permutations and the default *p* value < 0.05 was considered significant.

### 4.7. Protein–Protein Interaction Network Analysis

The protein–protein interaction network was created using the STRING (version 12.0) (https://cn.string-db.org), with a minimum required interaction score of 0.4. Cytoscape (3.10).3 was used for network visualization.

### 4.8. Mapping of Transcription Factor Binding Sites

The potential ESR1/2 and GR binding sites across the promoter region (1500 bp upstream and 500 bp downstream of the transcription start site) of the core genes were scanned by CiiiDER software (https://ciiider.erc.monash.edu/downloads.html (accessed on 17 January 2026)). JASPAR2020_CORE_vertebrates clustering was used as the transcription factor position frequency matrix, as described in our previous study [51].

### 4.9. Estimating Relative Cell-Type Proportions in Thymus Using Bulk RNA-Seq Data

To estimate the impacts of OVX, ADX, or both on thymic cell types, we applied IBERSORTx (https://cibersortx.stanford.edu) (accessed on 15 March 2025) to identify cell proportions using our bulk RNA-seq data for all samples. The signature matrix for the immune cells was built based on the mouse tissue expression profiles [52], and the signature matrix for adipocyte cells was generated based on transcriptome of representative genes for adipocytes [19]. The proportion of adipocytes and CD4^+^ thymocytes was then measured by deconvoluting gene expression levels of our bulk RNA-seq data based on the above two signature matrixes. The number of permutations for the statistical analysis was set as 1000.

### 4.10. Weighted Gene Co-Expression Network (WGCNA) Analysis

Weighted gene co-expression network analysis (WGCNA) was performed to identify modules of highly correlated genes using R. Co-expression network modules were identified using FPKM values, of which the genes with variation coefficients of averaged FPKM over 0.25 among all samples were analyzed. Subsequently, the correlation matrix was constructed by the “WGCNA” software package (version 1.73). The optimal soft threshold was chosen to convert the correlation matrix into an adjacency matrix, and a topological overlap matrix (TOM) was created from the adjacency matrix. The TOM-based phase dissimilarity metric was utilized to categorize genes with similar expression patterns into gene modules using average linkage hierarchical clustering. ME-based gene connectivity (kME) was calculated between the FPKM levels of each gene and the ME of modules. To identify co-expressing modules connected to thymic atrophy status, MEs were correlated with treatment groups. Modules with the strongest relevance to thymic atrophy status were selected as key modules for subsequent analysis.

### 4.11. Quantitative Analysis of mRNA Expression Using Quantitative PCR (qPCR)

Total RNA was isolated from thymus and adrenal gland tissues (Invitrogen Co., Carlsbad, CA, USA), according to manufacturer’s instructions. Quantitative and qualitative analyses of isolated RNA were assessed from the ratio of absorbance at 260 and 280 nm and agarose gel electrophoresis. A total of 500 ng RNA was converted into first-strand cDNA using a PrimeScript^®^ RT reagent kit with gDNA Eraser (TaKaRa Bio, Co., Ltd., Dalian, China). Quantitative real-time PCR was performed (in triplicate) on a CFX96 Real Time PCR detection system (Bio Rad, Hercules, CA, USA). The PCR reaction contained 1 μL cDNA, 500 nmol/L each of forward and reverse primers, and 2X SYBR^®^ premix TaqTM (TaKaRa Bio Co., Ltd.). Primer sequences of target and reference genes are shown in (Supplemental File S6). The PCR cycling conditions were as follows: initial denaturation at 95 °C (1 min), followed by 40 cycles of denaturation at 95 °C (5 s), annealing at 58–62 °C (25 s), and a final melting curve analysis (to monitor PCR product purity). The cycle threshold value was analyzed (CFX96 detection system) and transformed to a relative quantity using the standard curve method. A reference house-keeping gene (Gapdh) was measured for each sample; the relative abundance of the treatment groups was normalized to the sham control group.

### 4.12. Western Blotting

Protein lysates were prepared from the adrenal glands of mice by homogenization in SDS sample buffer (Bio-Rad, Hercules, CA, USA) containing β-mercaptoethanol (Sigma; St. Louis, MO, USA). Approximately 50 μg of total protein was resolved on a 4–20% Tris–glycine gel (Bio-Rad) and transferred onto a 0.2 μM nitrocellulose membrane (Bio-Rad), blocked with 1% BSA, and then incubated overnight with primary antibodies detecting CYP17A1 (1:1000; Abcam, ab134910; Cambridge, Cambridgeshire, UK) or GAPDH (1:5000; ABclonal Technology Co., Ltd., A19056; Woburn, MA, USA). Protein was then incubated with HRP-conjugated secondary antibodies (1:4000; ABclonal Technology Co., Ltd., AS014). The bands from Western blotting were visualized using an Immobilon Western Chemiluminescent HRP Substrate (Millipore, Burlington, MA, USA) and an ImageQuant LAS 4000 mini digital imaging system (GE Healthcare, Chicago, IL, USA). Band intensities were analyzed using Image J. The relative CYP17A1 protein levels were calculated as the ratio to that of GAPDH.

### 4.13. Statistical Analyses

Statistical analysis was performed using GraphPad Prism 9.2 software (La Jolla, CA, USA). All data underwent normality testing using Normality and Lognormality Tests before statistical analysis. Serum 17β-estradiol was log-transformed to normalize the distribution. Back-transformed means without correction were reported. All other data were normally distributed and did not require transformation before statistical analysis. Comparisons among groups were carried out via one-way ANOVA followed by Tukey’s test for multiple corrections. The values are presented as mean ± SEM. Significance is denoted by * for *p* < 0.05, ** for *p* < 0.01, and *** for *p* < 0.001.

## Data Availability

All data generated or analyzed during this study are included in this published article and its Supplementary Materials. The RNA sequencing raw data generated during the current study is available in the GSA database (https://bigd.big.ac.cn/gsa/browse; accession: CRA028484, accessed on 30 July 2025).

## Supplementary Materials

The following are available online at https://www.mdpi.com/article/10.3390/ijms27021022/s1.
