# Supplementary material for: Mechanical Insights into the Distinct Effects of Ovariectomy Versus Adrenalectomy on Age-Related Thymic Atrophy in Female Mice
Source: Int J Mol Sci. 2026 Jan 20;27(2):1022. doi: 10.3390/ijms27021022 (PMC12841801; doi:10.3390/ijms27021022)
Supplement: Supplementary file 1 [file ijms-27-01022-s001.zip › Supplemental File S3.pdf]

Supplemental Figures:

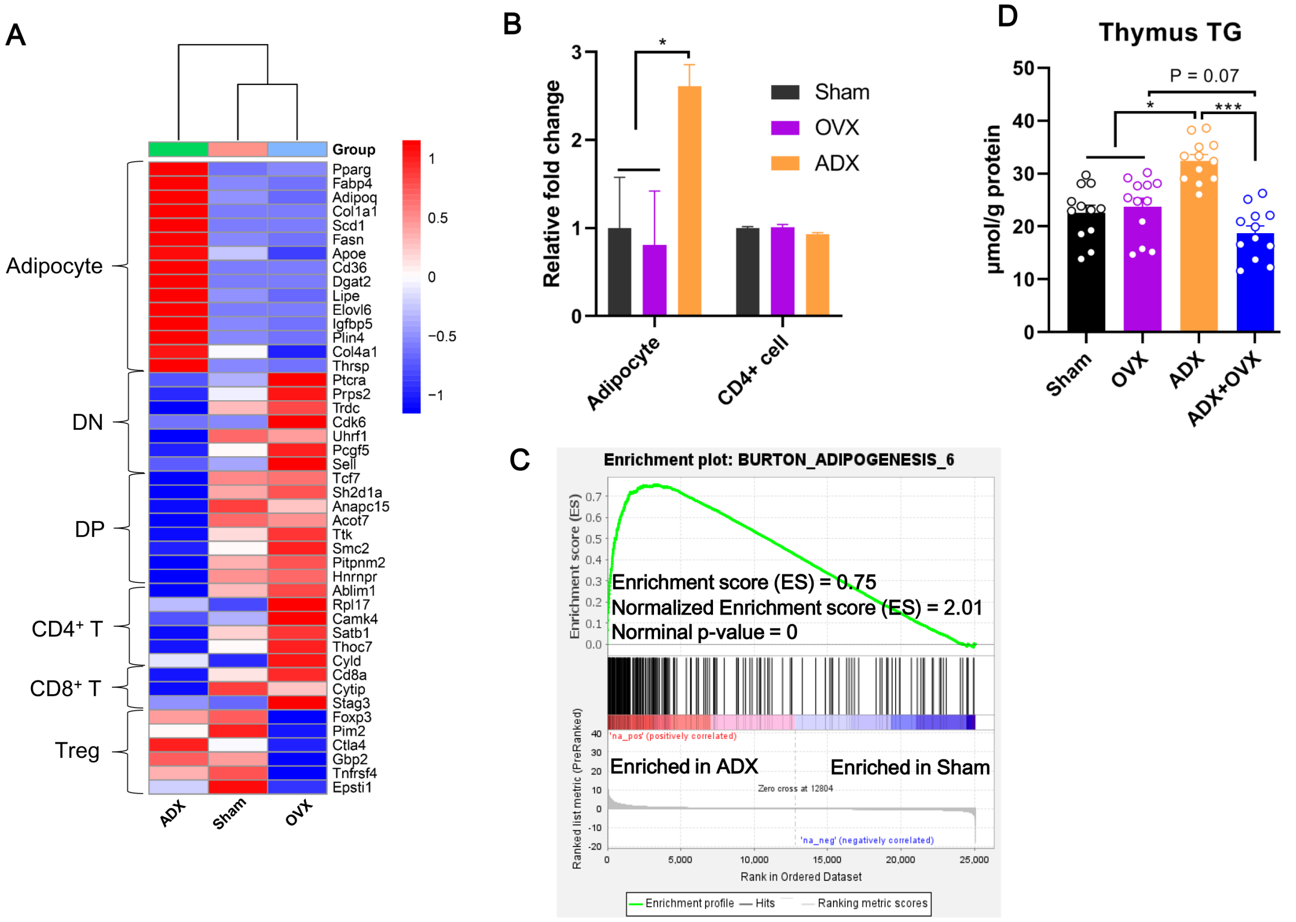

Figure S1. ADX promoted thymic adipose infiltration but blocked thymocyte proliferation in female mice. (A) The expression of cell marker genes for adipocyte, double-negative (DN), double-positive (DP), $CD4^{+}$ single-positive ($CD4^{+}$), and $CD8^{+}$ single-positive ($CD8^{+}$) T cells in thymus. (B) The estimated proportion of adipocytes and $CD4^{+}$ single-positive ($CD4^{+}$) T cells in Sham, OVX and ADX mice using CIBERSORTx algorithm. (C) Gene set enrichment analysis reinforced that ADX promoted adipogenesis in thymus
