# Supplementary material for: Mechanical Insights into the Distinct Effects of Ovariectomy Versus Adrenalectomy on Age-Related Thymic Atrophy in Female Mice
Source: Int J Mol Sci. 2026 Jan 20;27(2):1022. doi: 10.3390/ijms27021022 (PMC12841801; doi:10.3390/ijms27021022)
Supplement: Supplementary file 1 [file ijms-27-01022-s001.zip › Supplemental File S6.pdf]

qPCR Primer sequences for tissue genes

Gene	Genbank accession no.	Primer sequence (5′-3′)
Star	NM_011485.5	F: TCCCTCGCAGGACCTTGATCT<br>R: TGGATGGGTCAAGTTCGACG
Cyp11a1	NM_019779.4	F: GGCCCAATTTACAGGGAGAAG<br>R: CACCAGGGTACTGGCTGAAG
Cyp17a1	NM_007809.3	F: GAGGTGAAGAGGAAGATCCAAA<br>R: ATACGAAGCACTTCTCGGATAG
Nr3c1	NM_008173.4	F: AAAGAGACGAATGAGAGTCCTTGGA<br>R: GCTTGCAGTCCTCATTCGAGTTT
Pparg	NM_001127330.3	F: TGTTCGCCAAGGTGCTCCAG<br>R: TGAAGGCTCATGTCTGTCTCTGTC
Cdk1	NM_007659.4	F: CTCGGCTCGTTACTCCACTC<br>R: GCCACACTTCGTTGTTAGGAG
Cd74	NM_001042605.1	F: GCTGGATGAAGCAGTGGCTCTT<br>R: GATGTGGCTGACTTCTTCCTGG
Gapdh	NM_017008.4	F: CGGCAAGTTCAACGGCACAG<br>R: ACTCCACGACATACTCAGCAC

Abbreviations: Star, steroidogenic acute regulatory protein; *Cyp11a1*, cytochrome P450 family 11 subfamily A member 1; *Cyp17a1*, cytochrome P450, family 17, subfamily a, polypeptide 1; *Nr3c1*, nuclear receptor subfamily 3, group C, member 1(also called GR); *Pparg*, peroxisome proliferator activated receptor gamma; *Cdk1*, cyclin dependent kinase 1; *Cd74*, CD74 antigen; *Gapdh*, glyceraldehyde-3-phosphate dehydrogenase.
